# Supplementary material for: Molecular Classification and Tumor Microenvironment Characterization of Gallbladder Cancer by Comprehensive Genomic and Transcriptomic Analysis
Source: Cancers (Basel). 2021 Feb 10;13(4):733. doi: 10.3390/cancers13040733 (PMC7916565; doi:10.3390/cancers13040733)
Supplement: Supplementary file 1 [file cancers-13-00733-s001.pdf]

cancers

MDPI

Supplementary Material: Molecular Classification and Tumor Microenvironment Characterization of Gallbladder Cancer by Comprehensive Genomic and Transcriptomic Analysis

Nobutaka Ebata, Masashi Fujita, Shota Sasagawa, Kazuhiro Maejima, Yuki Okawa, Yutaka Hatanaka, Tomoko Mitsuhashi, Ayako Oosawa-Tatsuguchi, Hiroko Tanaka, Satoru Miyano, Toru Nakamura, Satoshi Hirano and Hidewaki Nakagawa

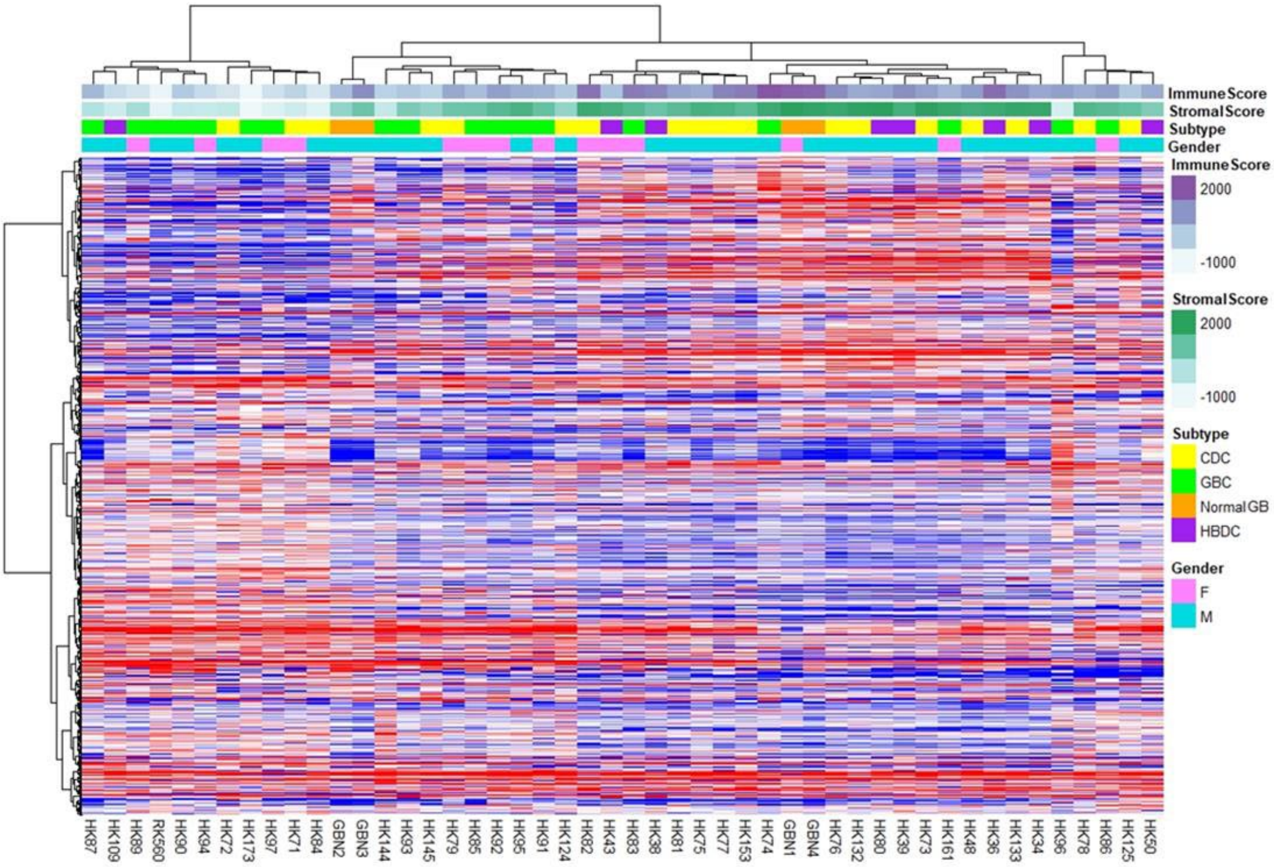

Figure S1. Hierarchical clustering analysis of protein-coding genes from 20 Gallbladder body and neck cancer (GBBCs), 16 Cystic duct cancer (CDSs), 8 Hilar bile duct cancer (HBDCs), and 4 normal gallbladders. Heatmap clustering of differentially expressed protein-coding genes. Subtypes were shown in each color (CDCs, yellow; GBBCs, green; HBDCs, purple; normal GB, orange). We failed to classify subtypes regardless of the anatomical site.

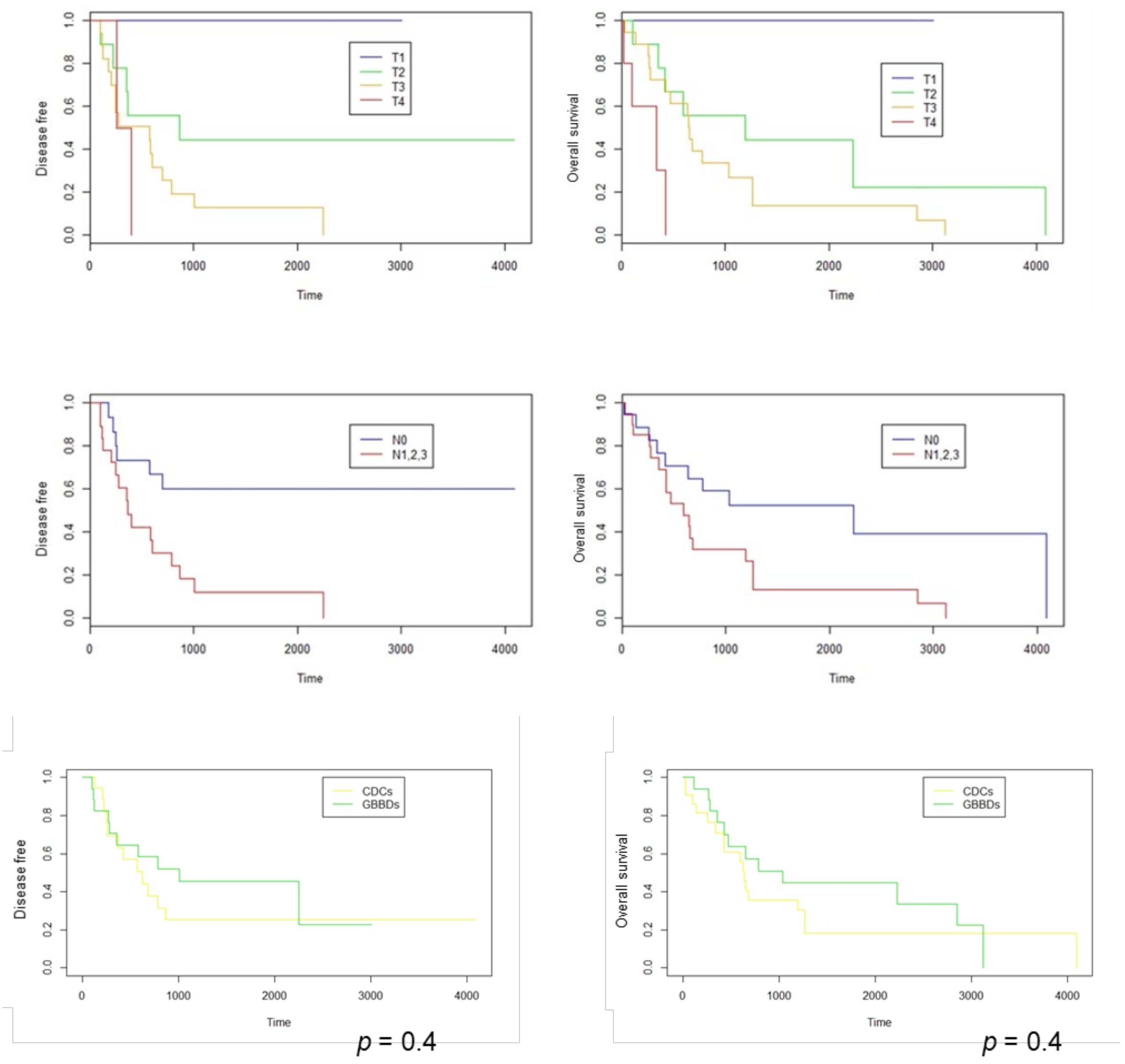

Figure S2. Disease-free survival (DFS) and overall survival (OS) of 36 GBCs, classified as pathological T, lymph node metastasis +/−, and GBBDs (Gallbladder body and neck cancer) /CDCs (Cystic duct cancer) in this study. The x-axis means days, and the y-axis means each ratio.

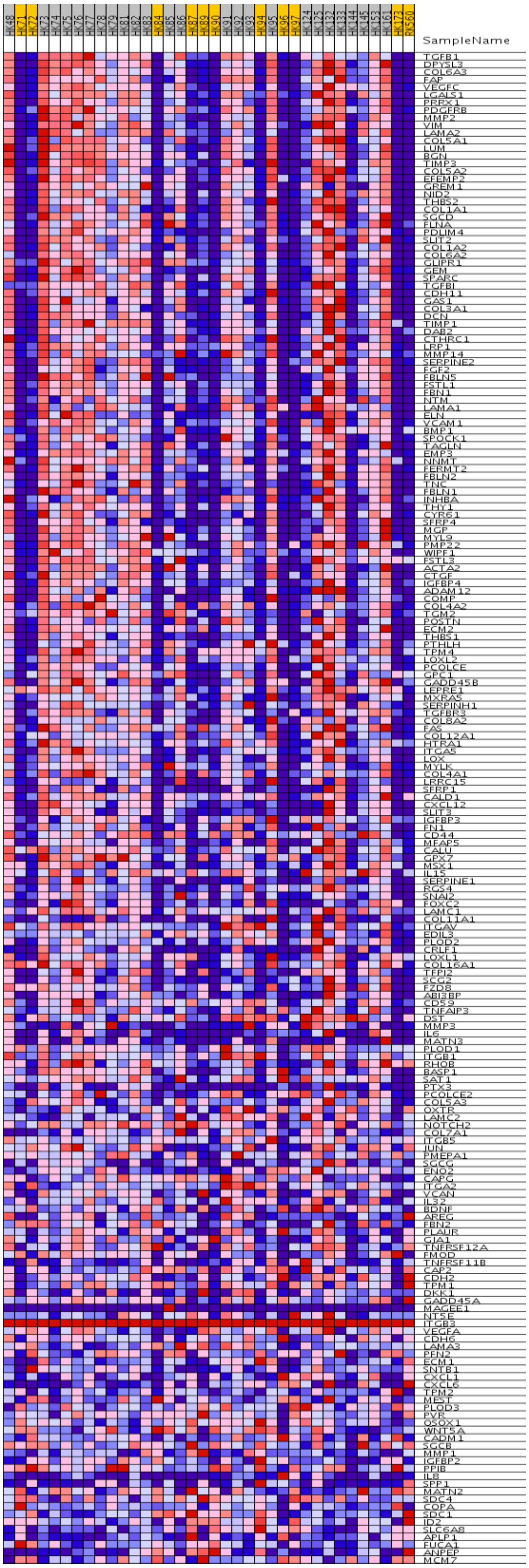

Figure S3. Heatmap shows the expression of EMT-related gene sets (198 genes) for GSEA and clustering. Samples marked as yellow were from Cluster A. High and low gene expression levels are shown as red and blue, respectively. This figure shows EMT-related gene sets that were significantly upregulated in Cluster B samples.

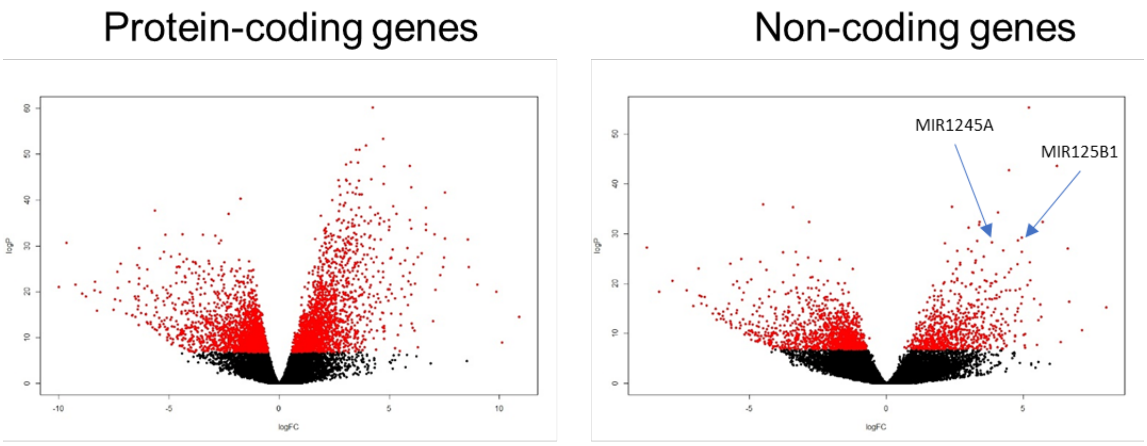

	Protein coding (n = 20,237)	Not protein (n = 35,528)
FDR<0.01	3,997	1,910

Figure S4. Comparison of the volcano plots of protein-coding and non-coding genes between the two clusters. The *x*-axis is log (fold change) and the *y*-axis is -log10 (*p*-value). The*MIR1245A* and*MIR125B1*were marked. The numbers of genes differentially expressed between Clusters A and B were 3997 protein-coding genes and 1910 non-coding genes (FDR < 0.01).

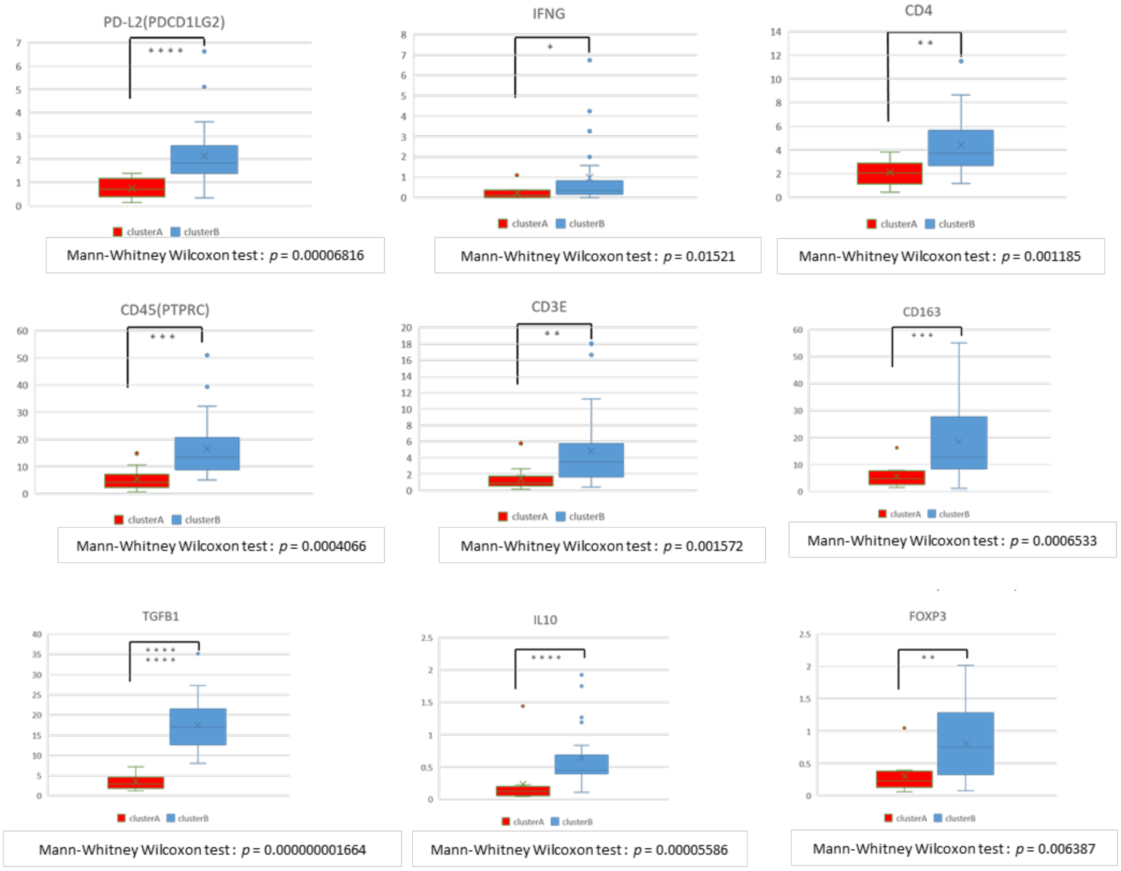

Figure S5. Comparison of the expression of immune genes (*PD-L2, IFNG, CD4, CD45, CD3E, CD163, TGFB1, IL10,* and*FOXP3*) between the two clusters in FPKM (* Mann-Whitney Wilcoxon test, $p< 0.05$). The *y*-axis shows FPKM of each gene.

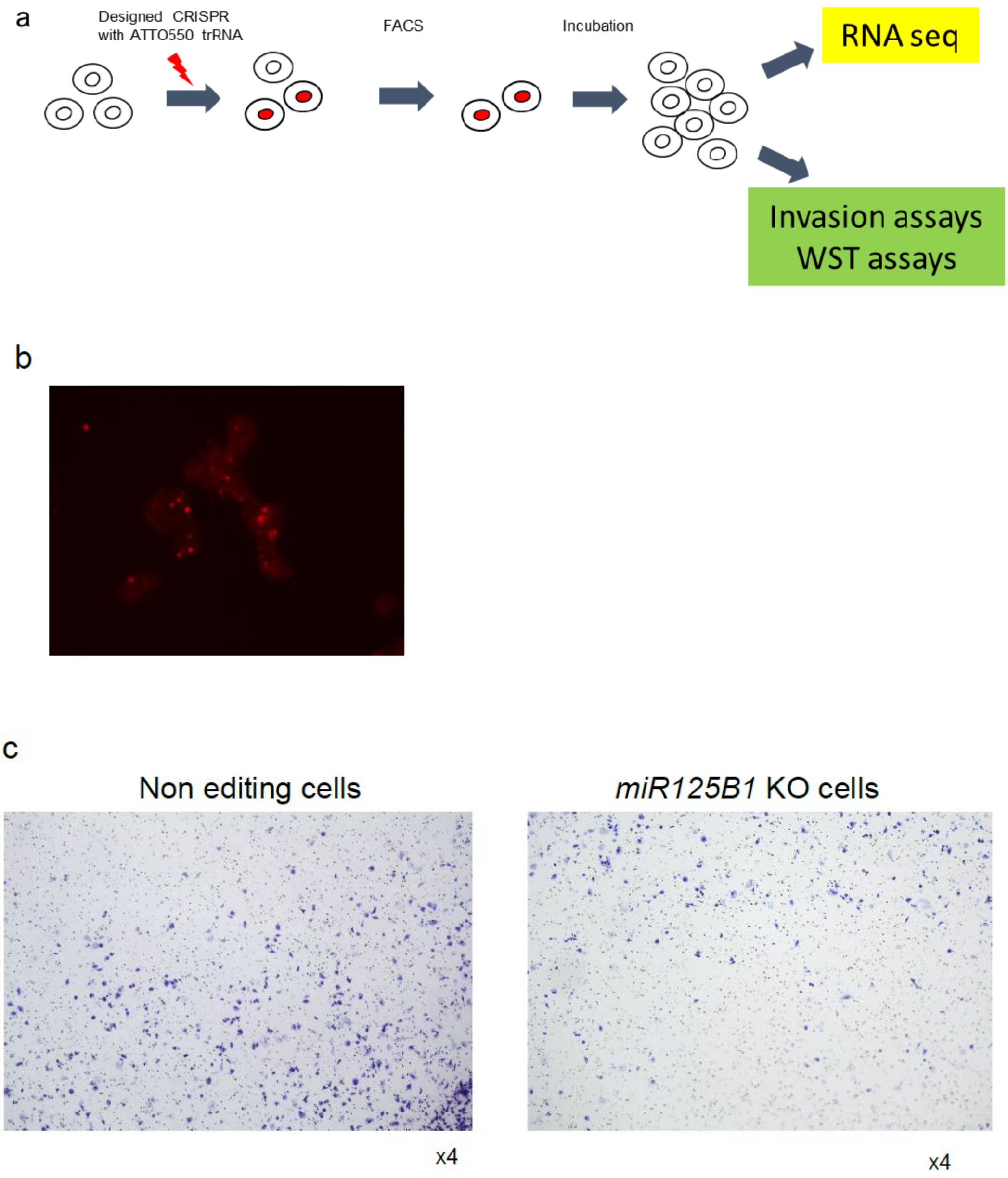

Figure S6. Knockout of *miR125B1* in GBC cell lines. (**a**) The schema of the knockout (KO) experiment in GBC cell lines. CRISPR protein and sgRNA with ATTO550-labeled trRNA were introduced by electroporation, and the edited cells were enriched by FACS. After incubation, the cells were analyzed by Matrigel invasion and cell viability assays. (**b**) Image of fluorescently labeled cells enriched after editing and FACS with a 10× objective lens. (**c**) Image of the membrane after the invasion assays. Left image, control NOZ cells; right image, *miR125B1*-KONOZ cells.

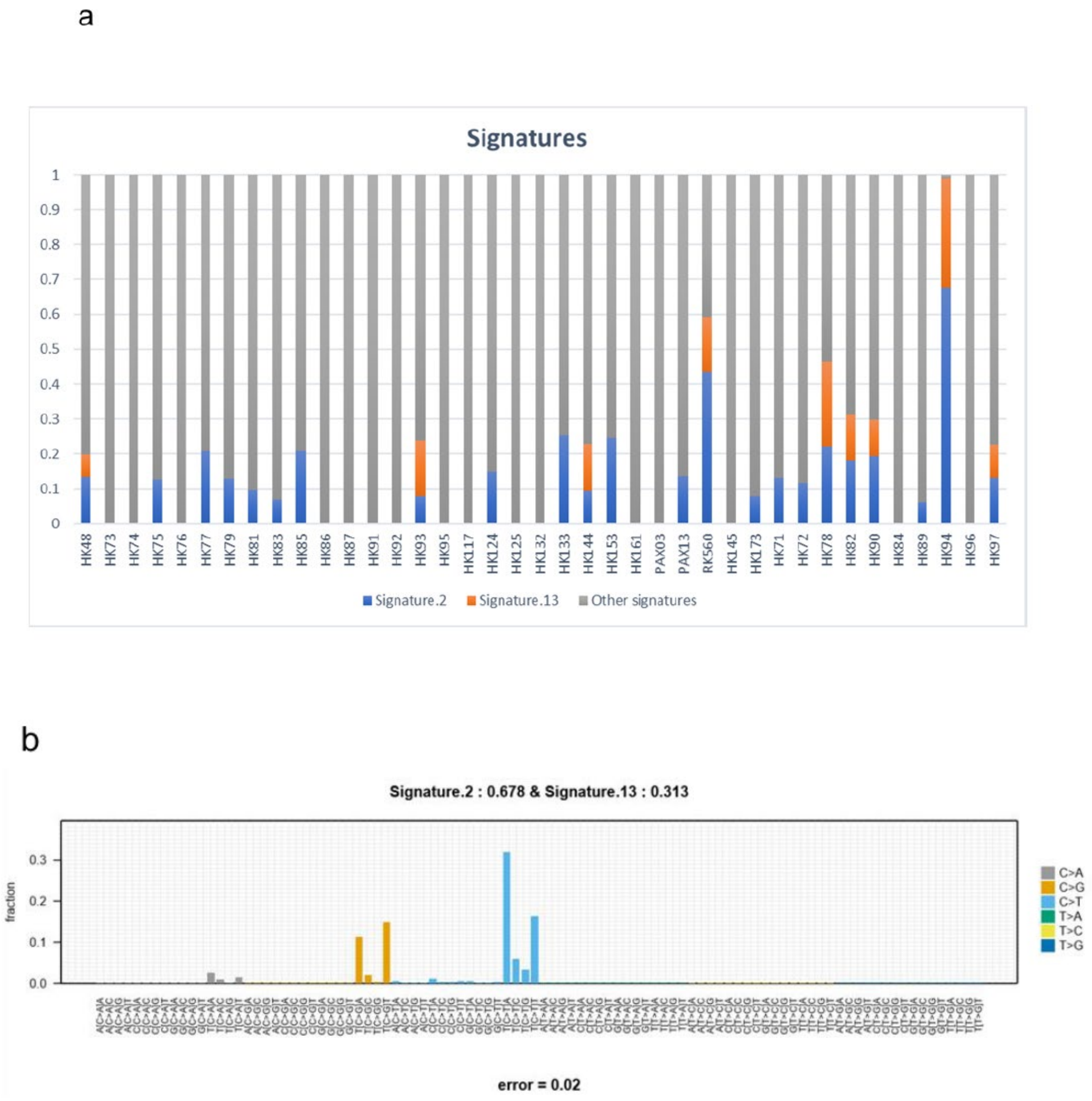

Figure S7. Mutational signatures of GBCs. (**a**) The abundance of COMIC APOBEC mutational signatures (blue: Signatures 2 and orange: Signature13) of 39 GBC samples, as analyzed using deconstructSigs (ver1.9.0, https://cran.r-project.org/web/packages/deconstructSigs/index.html, accessed on 1 December 2020). (**b**) Mutation signature pattern of HK96 with the largest mutation number and high APOBEC signature.

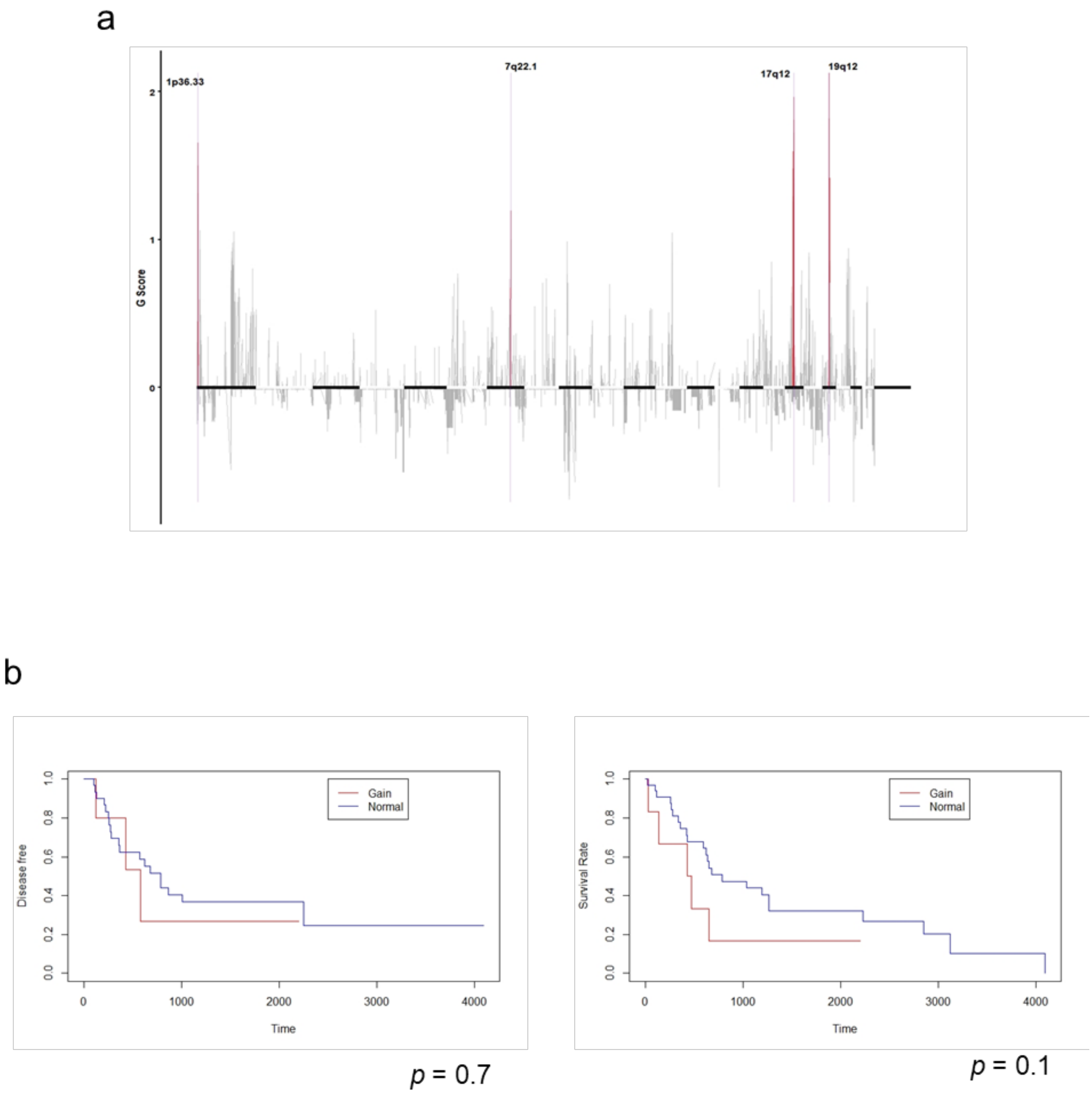

Figure S8. Copy number changes of GBCs. (**a**) Copy number analysis using segment file from 'Sequzenza' and GISTIC2. (**b**) Disease-free survival and total survival ratio of GBC patients with or without 19q12 gain.

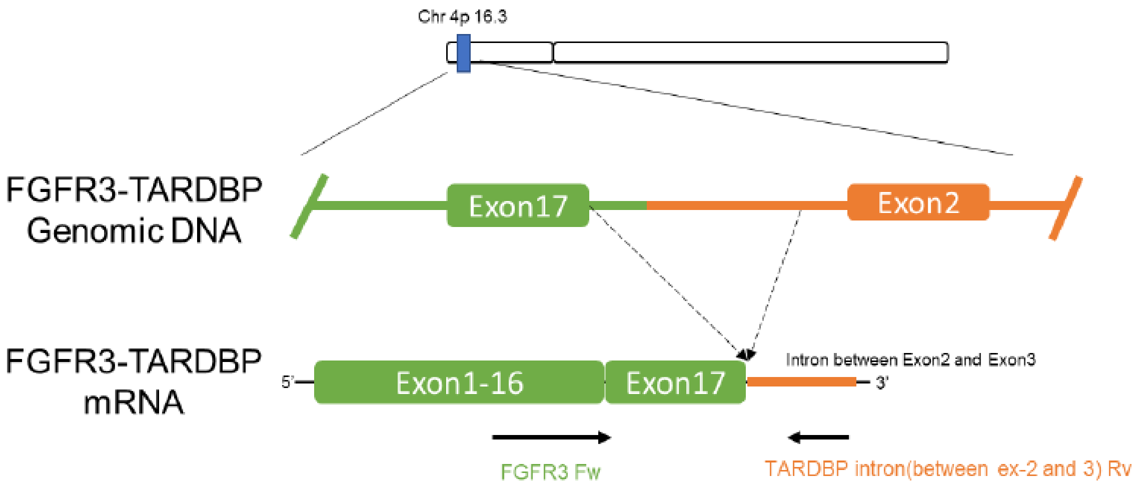

Figure S9. *TARDBP-FGFR3*gene fusion was detected in one sample (HK97). The scheme was estimated by IGV. It was created by a large deletion between *FGFR3*and *TARDBP* at chromosome 4p16.3. The expression level of *FGFR3*transcript in this tumor was much higher than that detected in other 35 cases (about 200 times in FPKM), indicating that it may constitutively activate *FGFR3*by changing its regulatory domain.

Table S1. Clinical features of 39 patients with GBC and 8 with HBDC in this study.

ID	Gender	Age	disease	pT	pN	Histology	RNAseq data	Tumor purity score
HK48	Male	83	CDC	2	1	mod+por	Available	0.523918825
HK71	Female	71	CDC	1	0	well	Available	0.925005055
HK72	Male	61	CDC	3	1	mod	Available	0.938118451
HK73	Female	58	CDC	4	0	mod	Available	0.471583686
HK74	Male	71	GBC	1	0	well	Available	0.29254934
HK75	Male	59	CDC	3	0	mod+por	Available	0.588185172
HK76	Male	66	CDC	3	0	NA	Available	0.460432001
HK77	Male	64	CDC	3	1	por	Available	0.482527123
HK78	Male	68	CDC	3	0	mod	Available	0.62587387
HK79	Female	71	CDC	3	1	mod+por	Available	0.670049628
HK81	Male	76	CDC	4	1	por	Available	0.606307532
HK82	Female	84	CDC	3	1	mod	Available	0.380080099
HK83	Female	68	GBC	2	1	mod	Available	0.491780621
HK84	Male	76	CDC	2	0	mod	Available	0.973868068
HK85	Female	76	GBC	2	1	por	Available	0.725428484
HK86	Female	81	GBC	3	0	adeno-squamous	Available	0.602990133
HK87	Male	72	GBC	1	0	mod	Available	0.83349456
HK89	Female	85	GBC	3	1	mod	Available	0.907945401
HK90	Male	82	GBC	2	0	well	Available	0.893610784
HK91	Female	72	GBC	3	1	well+mod	Available	0.683768336
HK92	Female	71	GBC	3	1	well	Available	0.595260436
HK93	Male	64	CDC	4	0	por	Available	0.782945765
HK94	Female	82	GBC	1	0	well	Available	0.930314751
HK95	Male	64	GBC	3	0	mod	Available	0.598960366
HK96	Male	63	GBC	3	1	adeno-endocrine	Available	0.844666913
HK97	Female	61	GBC	2	0	well	Available	0.962218252
HK117	Female	75	CDC	3	1	mod	Not available	Not available
HK124	Male	69	CDC	4	1	mod	Available	0.817237304
HK125	Male	77	CDC	4	1	mod	Available	0.740467467
HK132	Male	78	CDC	2	0	well	Available	0.501793375
HK133	Male	64	CDC	3	1	mod	Available	0.453367998
HK144	Female	73	GBC	3	1	NA	Available	0.91359976

HK145	Male	68	CDC	2	1	mod	Available	0.836544309
HK153	Male	70	CDC	2	1	mod	Available	0.502715066
HK161	Female	50	GBC	3	1	mod	Available	0.56573996
HK173	Male	80	CDC	1	0	well	Available	0.99412481
RK560	Male	51	GBC	2	0	mod	Available	0.988168454
PAX3	Male	54	GBC	3	1	adeno-squamous	Not available	Not available
PAX13	Male	65	CDC	3	0	well	Not available	Not available
HK34	Male	61	HBDC	4	0	mod	Available	Uncalculated
HK36	Male	72	HBDC	1	0	well	Available	Uncalculated
HK38	Male	72	HBDC	2	1	mod	Available	Uncalculated
HK39	Male	76	HBDC	2	2	mod	Available	Uncalculated
HK43	Female	65	HBDC	2	0	mod	Available	Uncalculated
HK50	Male	76	HBDC	2	0	por	Available	Uncalculated
HK80	Male	68	HBDC	3	1	well	Available	Uncalculated
HK109	Male	60	HBDC	2	1	well	Available	Uncalculated

GBBC: Gallbladder body and neck cancer, CDC: Cystic duct cancer, HBDC: Hilar bile duct cancer, pT: pathological T, pN: pathological N (Classification of biliary tract cancers established by the Japanese Society of Hepato-Biliary-Pancreatic Surgery: 3rd English edition). Tumor purity scores were calculated by ESTIMATE.

Table S2. Comparison of the pathological stages (pTand pN) between Cluster A/B (upper) by Fisher's exact test, and theirassociations with DFS and OS of GBC patients by Cox proportional hazard regression analysis (lower).

Factor	Cluster A	Cluster B	Fisher's Exact Test
Male: Female	7: 4	17: 8	$p = 1$
CDC (cystic duct): GBC (gallbladder)	3: 8	15: 10	$p = 0.1464$
pT (T1,2: T3,4)	7: 3	7: 18	$p = 0.0528$
pN (*N*−: *N*+)	8: 3	9: 16	$p = 0.07041$

Factor		HR	95% CI	*p-value*
Disease free survaival (DFS)	pT	1.7326	0.875–3.433	0.1152
	pN	3.3954	1.456–7.917	0.0047*
	Cluster A/B	2.519	1.182–5.368	0.0167*
Overall survaival (OS)	pT	2.648	1.266–5.540	0.0097*
	pN	1.3995	0.647–3.029	0.3936
	Cluster A/B	1.2896	0.6802–2.445	0.4358
				* $p < 0.05$

Table S3. Hallmark gene sets upregulated in Cluster B (FDR < 0.25).

NAME	FDR
EPITHELIAL_MESENCHYMAL_TRANSITION	0.001581567
IL2_STAT5_SIGNALING	0.013966793
ALLOGRAFT_REJECTION	0.014265655
INTERFERON_GAMMA_RESPONSE	0.014677468
IL6_JAK_STAT3_SIGNALING	0.015363473
ANGIOGENESIS	0.015474095
APOPTOSIS	0.015562979
TNFA_SIGNALING_VIA_NFKB	0.016412517
INFLAMMATORY_RESPONSE	0.01789831
MYOGENESIS	0.019249892
UV_RESPONSE_DN	0.021693027
KRAS_SIGNALING_UP	0.021736156
TGF_BETA_SIGNALING	0.028344875
HALLMARK_COAGULATION	0.028831769
HYPOXIA	0.029222561
HALLMARK_COMPLEMENT	0.02999398
APICAL_JUNCTION	0.031386394
HALLMARK_INTERFERON_ALPHA_RESPONSE	0.045963038
HALLMARK_HEDGEHOG_SIGNALING	0.04828798

HALLMARK_WNT_BETA_CATENIN_SIGNALING	0.050557055
HALLMARK_NOTCH_SIGNALING	0.072069064
HALLMARK_P53_PATHWAY	0.10861944
HALLMARK_APICAL_SURFACE	0.23982605

Table S4. The list of 24 genes significantly differentially expressed between Clusters A and B with an Figure 0. and a log fold change (FC) >3.9, including microRNAs.

Name	LogFC	FDR
WT1-AS	6.617791789	0.0000000004630000000000
LINC01050	6.216410121	0.0000000000000003620000
RP11-572C15.6	5.699776619	0.00000000000494
RP11-473L15.2	5.239561499	0.0000000048
RP1-79C4.4	5.196776962	0.0000000000000000000263
LINC00922	5.143063321	0.000000255
MIR125B1	4.947770833	0.0000000000683
RP11-716H6.1	4.934196867	0.000000000795
AC093850.2	4.873267411	0.00000046
RP11-170N16.2	4.804879186	0.00000000011
RP11-401O9.4	4.78588245	0.00000000652
SNORD113-5	4.721309503	0.000000618
RP11-401O9.3	4.55031903	0.000000016
PGM5P1	4.504826931	0.000000603
SNORD114-19	4.477585862	0.0000000282
RP11-820L6.1	4.467883412	0.000000000000000643
RP11-191N8.2	4.373821528	0.000000593
SNORD113-8	4.31886706	0.0000000711
RP11-335H2.2	4.270834919	0.00000000063
RP11-264F23.4	4.202812077	0.000000774
RP11-863P13.3	4.070876002	0.000000000000905
RP11-400N13.3	4.003583639	0.00000014
CTD-2270F17.1	3.998708651	0.0000000392
MIR1245A	3.979892033	0.000000133

Table S5. The ssGSEAscores of NOZ and G415 cells edited by CRISPR and score change ratio of the Hallmark signal activity. The change ratio of *miR125B*KO NOZ cells was calculated and the activity of EMT and inflammatory responsereduced after *miR125B*KO.

Name	NOZ_normal	NOZ_miR125B1_KO	Ratio NOZ KO	G415_normal	G415_miR125B1_KO	Ratio G415 KO
HALLMARK_BILE_ACID_METABOLISM	222.59	125.385	0.563	NA	NA	1.133
HALLMARK_KRAS_SIGNALING_UP	772.434	575.691	0.745	1,257.71	432.306	0.344
HALLMARK_INFLAMMATORY_RESPONSE	1,525.57	1,258.19	0.825	1,826.89	1,302.27	0.713
HALLMARK_IL6_JAK_STAT3_SIGNALING	910.715	764.67	0.84	2,700.09	2,453.91	0.909
HALLMARK_COMPLEMENT	3,455.88	3,062.81	0.886	4,418.37	3,668.86	0.83
HALLMARK_EPITHELIAL_MESENCHYMAL_TRANSITION	4,953.74	4,410.47	0.89	7,147.07	6,395.56	0.895
HALLMARK_INTERFERON_GAMMA_RESPONSE	2,789.91	2,528.60	0.906	2,527.21	1,902.16	0.753
HALLMARK_COAGULATION	2,944.08	2,704.28	0.919	4,376.55	3,363.90	0.769
HALLMARK_ESTROGEN_RESPONSE_EARLY	4,810.72	4,419.15	0.919	4,084.76	3,263.33	0.799
HALLMARK_APICAL_JUNCTION	6,605.51	6,068.78	0.919	6,637.65	6,356.53	0.958
HALLMARK_ESTROGEN_RESPONSE_LATE	4,821.02	4,469.59	0.927	4,923.10	4,358.86	0.885
HALLMARK_WNT_BETA_CATENIN_SIGNALING	3,281.39	3,064.55	0.934	3,186.97	2,678.38	0.84
HALLMARK_ANGIOGENESIS	4,095.76	3,847.83	0.939	3,524.51	2,719.77	0.772
HALLMARK_ALLOGRAFT_REJECTION	4,314.27	4,090.88	0.948	5,130.32	5,244.61	1.022
HALLMARK_UV_RESPONSE_UP	5,795.37	5,526.01	0.954	5,106.00	4,633.28	0.907
HALLMARK_NOTCH_SIGNALING	5,200.87	4,982.04	0.958	3,751.87	3,312.20	0.883
HALLMARK_IL2_STAT5_SIGNALING	4,680.09	4,530.84	0.968	4,465.87	4,228.31	0.947
HALLMARK_TNFA_SIGNALING_VIA_NFKB	4,736.97	4,587.32	0.968	5,310.40	5,341.53	1.006

HALLMARK_INTERFERON_ALPHA_RESPONSE	3,354.09	3,267.77	0.974	3,504.15	2,656.76	0.758
HALLMARK_TGF_BETA_SIGNALING	9,221.68	9,017.05	0.978	9,418.57	8,844.33	0.939
HALLMARK_P53_PATHWAY	7,222.81	7,071.57	0.979	6,505.37	6,478.15	0.996
HALLMARK_PANCREAS_BETA_CELLS	1,942.99	1,907.52	0.982	1,415.04	370.996	0.262
HALLMARK_MYC_TARGETS_V2	9,822.78	9,683.92	0.986	9,285.38	9,502.62	1.023
HALLMARK_GLYCOLYSIS	7,796.36	7,729.90	0.991	7,883.41	7,431.00	0.943
HALLMARK_DNA_REPAIR	8,165.15	8,127.98	0.995	8,456.59	8,429.73	0.997
HALLMARK_APOPTOSIS	7,417.40	7,384.13	0.996	7,442.25	7,166.26	0.963
HALLMARK_CHOLESTEROL_HOMEOSTASIS	8,112.91	8,090.59	0.997	7,863.50	8,079.44	1.027
HALLMARK_UNFOLDED_PROTEIN_RESPONSE	9,746.65	9,728.81	0.998	9,767.40	9,669.59	0.99
HALLMARK_REACTIVE_OXYGEN_SPECIES_PATH-WAY	9,425.73	9,413.47	0.999	9,656.52	9,420.58	0.976
HALLMARK_MYC_TARGETS_V1	10,744.30	10,742.97	1	10,681.39	10,697.25	1.001
HALLMARK_G2M_CHECKPOINT	9,916.84	9,918.66	1	9,900.77	9,788.62	0.989
HALLMARK_PI3K_AKT_MTOR_SIGNALING	7,946.81	7,955.51	1.001	8,049.42	7,840.69	0.974
HALLMARK_HEDGEHOG_SIGNALING	1,203.14	1,204.89	1.001	2,090.39	2,329.30	1.114
HALLMARK_KRAS_SIGNALING	4,969.65	4,978.95	1.002	5,197.99	4,444.49	0.855
HALLMARK_ANDROGEN_RESPONSE	7,736.95	7,754.94	1.002	7,311.51	7,092.72	0.97
HALLMARK_MTORC1_SIGNALING	9,519.86	9,548.71	1.003	9,472.06	9,394.95	0.992
HALLMARK_E2F_TARGETS	10,022.18	10,055.19	1.003	10,174.16	10,107.40	0.993
HALLMARK_OXIDATIVE_PHOSPHORYLATION	9,867.71	9,901.83	1.003	9,539.71	9,567.55	1.003
HALLMARK_HYPOXIA	7,710.40	7,762.74	1.007	8,176.84	8,033.99	0.983
HALLMARK_ADIPOGENESIS	6,932.56	6,984.20	1.007	6,513.15	6,340.19	0.973
HALLMARK_PROTEIN_SECRETION	8,819.26	8,888.66	1.008	8,895.40	8,630.77	0.97
HALLMARK_MITOTIC_SPINDLE	8,740.81	8,832.47	1.01	8,807.34	8,659.20	0.983
HALLMARK_SPERMATOGENESIS	1,366.74	1,386.66	1.015	2,889.48	2,719.36	0.941
HALLMARK_PEROXISOME	6,730.41	6,839.66	1.016	6,586.77	6,496.25	0.986
HALLMARK_UV_RESPONSE_DN	6,562.83	6,670.50	1.016	7,624.19	7,439.28	0.976
HALLMARK_FATTY_ACID_METABOLISM	7,127.32	7,259.87	1.019	7,235.17	7,307.46	1.01
HALLMARK_HEME_METABOLISM	4,321.30	4,450.65	1.03	4,551.92	4,130.09	0.907
HALLMARK_APICAL_SURFACE	1,374.20	1,427.43	1.039	231.421	530.879	2.294
HALLMARK_KRAS_SIGNALING_DN	NA	NA	1.049	NA	NA	1.018
HALLMARK_XENOBIOTIC_METABOLISM	3,259.03	3,560.51	1.093	4,314.23	3,560.13	0.825
HALLMARK_MYOGENESIS	1,262.27	1,609.37	1.275	2,922.44	2,566.65	0.878
HALLMARK_UV_RESPONSE	NA	NA	1.491	NA	NA	1.114

Table S6. Oligonucleotides sequences for sgRNA(upper)and PCR(bottom)primers used in this study.

Gene	Number	Target Sequence	GC	TM(°C)	Plus or Minus	Position（RNA）
MIR1245A	①	ATACTCTTTAAGTGATCTAAAGG	25%	59.36	plus	36-58
	②	CCTTTAGATCATCTGATGTTGAA	30%	61.41	minus	14-36
MIR125B1	①	ATGTTTACCGTTTAAATCCACGG	30%	62.53	plus	36-58
	②	CCCTAACTTGTGATGTTTACCGT	35%	65.15	minus	24-46

Primer	Product Length	Sequence	Tm
MIR1245A-F	176	CTCAGGTAATAACAGAGCCTTGA	57.66
MIR1245A-R		TGCTTTCTTTTCTTGTAATGCTGA	57.61
MIR125B1-F	181	ACCTCGAACAGAAATTGCCT	57.43
MIR125B1-R		AATTCCACCAAATTTCCAGGATG	57.57
